# Association Between Orthorexia and Plant-Based Diets—Is There a Vicious Cycle?

**DOI:** 10.3390/nu17081337

**Published:** 2025-04-13

**Authors:** Patrycja Szulc, Kaja Willich, Patrycja Gogga

**Affiliations:** Division of Food Commodity Science, Department of Clinical Nutrition, Faculty of Health Sciences, Medical University of Gdańsk, ul. Dębinki 7, 80-211 Gdańsk, Poland; patkaszulc@gumed.edu.pl (P.S.); kaja.willich@gumed.edu.pl (K.W.)

**Keywords:** plant-based diets, vegan, vegetarian, orthorexia nervosa, healthy orthorexia, eating disorders

## Abstract

Nowadays, social media and rapidly changing dietary trends encourage people to constantly—often excessively—control their diet, which leads to an increased risk of developing eating disorders, including orthorexia nervosa (ON). At the same time, more and more people reduce or give up consumption of meat and other animal products, adopting different types of plant-based (vegetarian) diets. The following paper aimed to demonstrate the significant similarities between orthorexia and plant-based diets and to highlight the necessity of developing new or adapting existing diagnostic tools for orthorexia considering the lifestyle characteristics of vegetarians, especially vegans. It remains unclear whether vegetarianism increases the risk of developing ON or whether the development of ON increases the chances of switching to a plant-based diet. However, based on the available literature, a positive association can be shown between the two. On the one hand, a vegetarian diet may be a cover for ON, but on the other, vegetarians, as a group avoiding specific food products, may be wrongly perceived as disordered. Thus, it is important to distinguish ON from the so-called healthy orthorexia (HO), which is a non-pathological interest in healthy eating, to avoid overdiagnosis and stigmatization of people using healthy alternative diets, including plant-based diets, since the mere fact of following them does not constitute evidence of orthorexia.

## 1. Introduction

People’s appearance affects their sense of self-worth. There is a belief that having a certain figure can make them fulfilled and happy human beings. The canons of beauty have changed over time. For instance, in the past, boyish, curvy, or athletic figures were desired in women. Currently, slim figures of women and athletic and muscular bodies of men are glorified. A “perfect” appearance has become a necessity, a feature that determines intelligence and control over life [1]. The pursuit of a desired body image is etiologically related to the pressure of society, which results from contemporary standards of beauty. Unachievable perfection increases criticism of one’s own body image and the risk of certain triggers, such as depression or undertaking restrictive diets, contributing to the development of eating disorders [2]. 

Eating disorders are behavioral states characterized by serious and persistent disturbances of eating practices that affect physical, mental, and social health [3]. They are influenced by many factors: biological, personal, family, and socio-economic [2]. Nowadays, social media and wellness culture encourage people to constantly analyze their diet, often resulting in a worsening of one’s relationship with food. Giving up specific food products and so-called clean eating—consuming only foods regarded as healthy, e.g., primarily unprocessed [4]—usually begins as an attempt to achieve optimal health through attention to diet but may lead to various eating disorders, including orthorexia nervosa (ON), and ultimately to malnutrition and low quality of life [5].

ON is the ideology of eating “only the right food”. It involves counting calories, eliminating “fattening” products, and generally consuming a very modest group of foods. It can manifest itself with obsessive thoughts about food and constant meal planning, and people struggling with this disorder do not take pleasure in eating because they are afraid of it. Possible health consequences of ON are mineral and vitamin deficiencies, and—as a consequence—osteoporosis, hypertension, and anemia [6]. 

Limiting or excluding meat and other animal products is becoming increasingly popular, especially among young people. Despite the documented health benefits of adopting such dietary patterns, studies show a significant relationship between them and eating disorders—more than half of patients with eating disorders report that they began following various types of vegetarian diets after the onset of their disordered eating [7].

The following review aimed, firstly, to explore the hypothesis that ON and plant-based diets are interrelated and, secondly, to show that diagnostic tools currently used for ON are not applicable to vegetarians regarding their specific eating habits. Particular attention was also paid to the distinction between orthorexia and a non-pathological interest in healthy eating.

## 2. Methodological Approach and Search Strategy

The following paper is a narrative review. A literature search on the relationship between plant-based diets and orthorexia was conducted in PubMed and Google Scholar using the following keywords: “orthorexia nervosa”, “plant-based diets”, “vegetarianism”, “veganism”, and “eating disorders”. After an initial screening of the abstracts, only studies closely related to the topic were chosen, i.e., assessing the incidence of ON among vegetarians and discussing the possible link between this eating disorder and plant-based diets. Basically, studies published between 2015 and 2024 in English and Polish were included; however, literature addressing definitions and the validation of questionnaires used as diagnostic tools as well as papers published earlier were excluded.

## 3. Plant-Based Diets

More and more people are deciding to give up meat and other animal products. There is a wide range of plant-based (vegetarian) diets—from less to more restrictive: lacto-ovo-vegetarianism (excludes meat and fish), ovo-vegetarianism (excludes meat, fish, and dairy), lacto-vegetarianism (excludes meat, fish, and eggs), pescetarianism (excludes only meat), and veganism (excludes all animal products). There are also so-called pseudo-vegetarian diets, namely semi-vegetarianism and flexitarianism, in which meat consumption is partially limited [8]. 

According to the largest dietetic associations (Academy of Nutrition and Dietetics, Dietitians of Canada, British Dietetic Association), properly balanced vegetarian diets (including vegan) are healthy and can be an appropriate choice for any stage of life (during pregnancy and lactation, in children, and in the elderly) and in athletes [9]. Additionally, the use of plant-based diets can bring numerous health benefits. Compared to meat eaters, vegetarians (especially vegans) have lower body mass and BMI [10]. Furthermore, plant-based diets can reduce the risk of diet-related diseases, including hypertension, type 2 diabetes, or some cancers [8]. However, followers of these diets are prone to deficiency of certain nutrients, namely vitamin B_12_ (supplementation of which is essential in a strict plant-based diet), vitamin D, calcium, iodine, iron, zinc, selenium, vitamin A, omega-3 fatty acids, and protein [9,10,11].

Usually, adopting a plant-based diet is an attempt to modify eating habits, during which animal-based foods and highly processed foods are replaced—whenever possible—with raw, unprocessed, or minimally processed plant-based products. There are several reasons for switching to a vegetarian diet, including health, ethical, or environmental motives [12].

## 4. Similarities Between Plant-Based Diets and Orthorexia Nervosa

**Figure 1:**
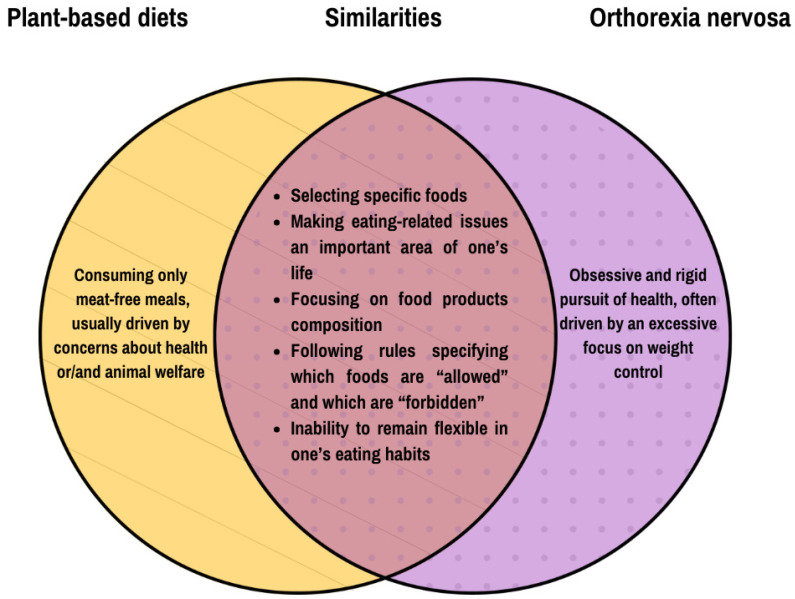
Similarities between plant-based diets and orthorexia nervosa. Based on [13].

ON shares several features and dieting behaviors with vegetarianism, such as restricting food intake according to specific dietary rules and an inability to be flexible in one’s eating habits (Figure 1) [13]. Thus, adhering to these diets involves restrictions resembling cognitive restraint—the intention to continually, consciously control food intake to maintain or lose weight [14]—often used as an indicator of pathological eating behaviors [15]. However, it may not be applicable to vegetarians (especially vegans), as their avoidance of animal products is not necessarily indicative of disordered eating [16]. In fact, a study on vegetarians and vegans showed that both groups had lower cognitive restraint, emotional eating, and uncontrolled eating compared to omnivores, but on the other hand, they exhibited more behaviors associated with ON, such as a stronger focus on healthy eating, greater knowledge of it, and a heightened sense of wellbeing related to healthy food choices [15].

Nevertheless, recent studies suggest that ON is associated with impaired cognitive functions, and plant-based diets could be a contributing factor [13]. Consequently, obsessive thoughts of food—a key feature of ON—appear to be more prevalent among vegetarians than non-vegetarians [17], potentially putting them at a higher risk of developing orthorexic tendencies or the disorder itself [15,18,19].

## 5. Healthy Orthorexia

**Figure 2:**
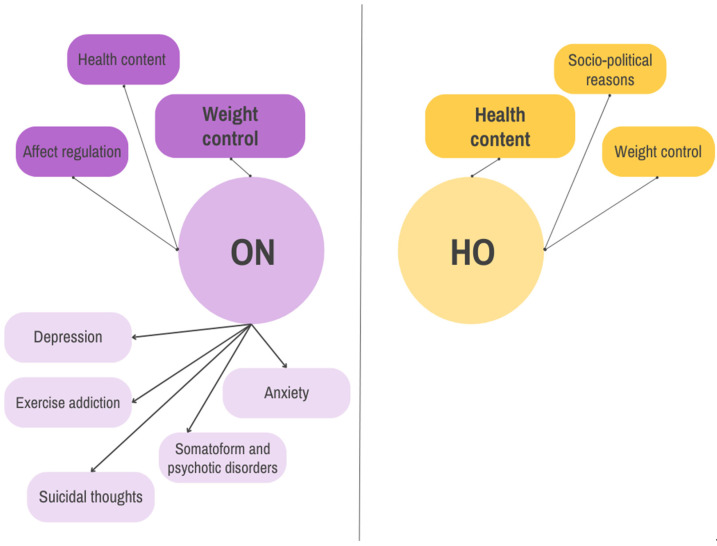
Motives for food choices and their psychopathological consequences in two types of orthorexia. Bigger font size indicates predominant motives. ON—orthorexia nervosa; HO—healthy orthorexia. Based on [21,24].

Some studies suggest that orthorexia may also be a non-pathological interest in healthy eating, which is called a healthy orthorexia (HO) [20]. The bidimensional concept of orthorexia includes both pathological (ON) and non-pathological (HO) aspects of interest in healthy eating, which appear to vary significantly (Figure 2). In the case of ON, the motive showing the strongest association is weight control. Health content shows negligible and statistically insignificant relationship with ON. In the case of HO, the strongest association is observed with health content, followed by weight control and socio-political reasons [21]. It should be emphasized, though, that constant reductions in acceptable foods, resulting in a diet that includes only a small number of products considered edible, may not necessarily lead to a decrease in overall calories intake, which—in fact—may even be increased in subjects with ON [22]. Moreover, despite their apparent preoccupation with healthy eating, individuals with ON often exhibit relatively unhealthy eating habits and other undesirable lifestyle behaviors, such as increased screen time or increased smoking and alcohol consumption compared to healthy subjects [23]. 

## 6. Diagnostic Criteria for Orthorexia Nervosa

ON is a relatively newly described eating disorder, and despite the constantly increasing awareness and public interest in this issue, it cannot be clearly classified as an eating disorder or a mental disorder. The available literature discusses whether ON should be considered a separate disorder, a variation on other disorders, or a cultural attitude [25,26,27]. It should be emphasized that ON is not included in the current Diagnostic Criteria for Mental Disorders (DSM-5-TR) or in the International Statistical Classification of Diseases and Related Health Problems (ICD-11) [25,26,27]. In addition, diagnosing ON may be difficult due to the lack of standard diagnostic criteria in the main psychiatric classification systems. It is also important to distinguish ON from other eating disorders (e.g., symptoms and behaviors typical for ON may also be characteristic for anorexia nervosa). Finally, it is crucial to differentiate between HO and ON to recognize where a care of one’s diet quality ends and a pathological obsession—potentially leading to suffering and health damage—begins [26,27] and to avoid overdiagnosis and stigmatization of all alternative healthy diets (including plant-based diets) [26]. Nevertheless, the mere fact of reducing meat for ethical reasons should not be a diagnostic criterion for ON [28].

At the moment, despite many proposals for diagnostic criteria for ON, no uniform standard or consensus has been established [27]. However, proposed criteria for ON usually include the following features:

- Obsessive focus on healthy eating (rigid rules and restrictions, excessive attention to the nutritional value of meals, as well as the quality and purity of food) worsening everyday functioning;
- Emotional disorders (e.g., anxiety, fear, etc.) resulting from failure to follow the rigorous dietary rules imposed on oneself;
- Psychosocial problems in various areas of life (e.g., work- or school-related);
- Malnutrition and weight loss [26,27].

Selected psychometric tools for diagnosing ON are presented in Table 1. The most used are the ORTO-15 questionnaire and the Bratman Orthorexia Test (BOT) [27]. 

## 7. Two Sides of the Cycle

**Figure 3:**
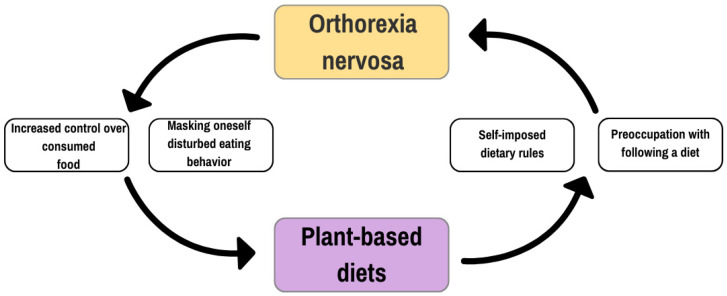
The vicious cycle of the relationship between orthorexia nervosa and plant-based diets. It is presumed that individuals preoccupied with strict dietary rules may develop orthorexic tendencies, while those with orthorexia may use plant-based diets to justify or mask abnormal eating behaviors. The cycle reinforces increased control over food intake and perpetuates dietary restrictions.

Half of patients with anorexia nervosa declare that they refuse to eat red meat because it is perceived as too caloric, distasteful, or anxiety-provoking. On the other hand, problematic eating behaviors developed after switching to a vegetarian diet, in the form of significant restrictions on the amount, frequency, or variety of food consumed, have also been observed [44]. Other studies indicate that 50% of anorexic patients follow a vegetarian diet [45], compared to 10% in the general population [46]. It remains unclear whether vegetarianism increases the risk of developing eating disorders or whether having an eating disorder increases the chances of choosing plant-based dietary pattern [47]. It cannot be ruled out that the pathology is perpetuated in a vicious cycle, in which restriction begets restriction [48] (Figure 3).

### 7.1. Plant-Based Diets as a Cover for Orthorexia Nervosa

Currently, there is an increased prevalence of vegetarianism (from 1% to 9%), especially in developed countries [45]. The reasons for choosing this dietary pattern vary depending on the study. One indicated that health/nutrition (37.5%) is the most common reason, followed by weight control (18.8%) and ethics towards animals (14.6%) [49]. In another study, the most common reasons were taste preferences (58.1%), followed by a healthier diet (19.4%) and weight control (9.6%) [50].

Conscious eating is associated with long-term health benefits but also with a sense of responsibility, which in some people can lead to an obsession with healthy eating, characterized by excessive control over food intake, forced behaviors, and self-control [51]. Some research suggested that use of the plant-based diets may be associated with a disturbed body image and complexes regarding body weight and figure that do not correspond to the contemporary canons of beauty [45]. Moreover, studies have shown that women who follow a vegetarian diet may be more likely to demonstrate disturbed eating attitudes and behaviors than women who follow an omnivore diet as well as men. It was also found that following certain diets or dietary rules, such as different types of vegetarian diets, is associated with orthorexic eating patterns [13]. 

Many people with disturbed eating habits and using (currently or in the past) a vegetarian diet admit that they adopted it because they wanted to increase control over the consumed food. In a patient with eating disorders and following a vegetarian diet, there is a high probability that the diet is a way of restricting one’s food consumption as a part of the pathological eating behavior [52]. Vegetarianism may be a widely accepted way to limit food intake to control or reduce body weight while concealing pathological eating behaviors [53]. Since plant-based diets are characterized by a high intake of products such as vegetables, whole grains, legumes, and fruits, they have a positive effect on health, as they are high in fiber, bioactive compounds, and micro- and macro-elements and low in saturated fats and simple sugars [54]. This affects the perception of plant-based diets as low-calorie and low-fat, which on the one hand is good for general health [46] but on the other may be a strategy for avoiding food to control body weight [48]. It has been noted that eating disorders occurred more frequently in people who declared using a vegetarian diet for this very purpose [17].

With the right expertise and meal planning, there is no reason why a vegetarian—including vegan—diet cannot be well balanced and sufficient to meet the nutritional needs of any individual [55]. However, a person with ON exhibits inflexible eating behaviors, ranging from a focus on eating organic or raw foods to a complete avoidance of all foods perceived as unhealthy, which can lead to a disturbed relationship with food and malnutrition [45]. In one study, participants most frequently named plant-based foods (30%), organic/natural/non-GMO foods (18%), and protein foods (16%) as important components of “clean” diets [56]. Furthermore, two-thirds of people with a history of eating disorders reported that their choice of vegetarianism was associated with their eating disorders because it allowed them to reduce calorie intake and to increase their sense of control over consumed food [52].

Through avoidance and ritualization—perceiving vegan foods as healthy—people with ON experience temporary relief from anxiety while perpetuating intrusive thoughts about health and food [23]. 

The higher prevalence of eating disorders in vegetarians suggests that plant-based diets may be used to justify avoidance of food and to mask restrictive eating patterns used for weight control [13]. For a patient with an eating disorder, declaring being a vegetarian for animal rights reasons, for example, seems more socially acceptable and less uncomfortable than explicitly disclosing weight loss as a motive [48]. 

### 7.2. Plant-Based Diets as a Path to Orthorexia Nervosa

Although there is an increasing number of studies on vegetarianism, only few have examined the relationship between the use of these dietary patterns and a tendency to orthorexia disorders [13,28,47]. It has been suggested that vegetarianism may be a factor triggering and maintaining eating disorders, but the evidence supporting this thesis is ambiguous [53,57].

The results of studies assessing the incidence of ON among vegetarians are divergent—some have indicated a significant link between the use of plant-based diets and this disorder [28,47,58,59,60], while others have found no such association [36,61,62]. The discrepancy in results may be due to several reasons, including different definitions of types of vegetarianism or the use of different methods for diagnosing ON [28] and their not being adjusted to plant-based diets users; for obvious reasons, the dietary attitudes and behaviors of vegetarians differ from non-vegetarians, and this may be wrongly perceived as pathology [63].

Missbach et al. [58], in a sample of 1029 people, found that different vegetarian dietary patterns were associated with the occurrence of ON. Similarly, Kozik and Całyniuk [60], in a sample of 514 students from the First High School in Tychy, including 34 individuals following a vegetarian diet, found that while gender was not a significant risk factor for ON, adherence to a vegetarian diet was: more than a half of the vegetarians were at risk for ON compared to 30.2% of non-vegetarians [60]. Also, Dell’Osso et al. [64], in a sample of 2130 people from the academic community of the University of Pisa, found that vegetarians showed a significantly higher rate of ON symptoms than people on a typical diet. Moreover, women who obtained lower (more pathological) ORTO-15 scores were characterized by a lower BMI, were more often underweight, and were more often vegetarians [64].

Reynolds et al. [59] compared the tendency toward ON between vegans, vegetarians (using lacto-, ovo-, and lacto-ovo-vegetarian diets and different types of pseudovegetarian diets), and people who did not exclude any product groups. It was found that vegans and vegetarians had significantly more orthorexic tendencies than non-vegetarians. A particularly high incidence of ON was observed in the vegan group: 42.9% in dietary vegans and 30.8% in lifestyle and dietary vegans compared to 11.8% in omnivores. Furthermore, it was shown that people with greater orthorexic tendencies chose food for weight control and ethical reasons significantly more often than people with fewer orthorexic tendencies [59]. 

On the other hand, some studies do not support the thesis that plant-based diets and ON are related. Çiçekoğlu and Tunçay [61] examined the link between ON and dietary habits, comparing 31 vegans/vegetarians with 31 non-vegans/non-vegetarians. Using standardized questionaries (ORTO-11, EAT-40, and MOCI), researchers found no significant differences in ON tendencies or obsessive symptoms between the groups. Vegans/vegetarians adopted their diet primarily for ethical reasons and not due to a pathological focus on healthy eating. The authors concluded that neither vegetarianism nor veganism are inherently associated with ON and called for further research on that subject [61]. In a study investigating the relationship between social media usage and the risk of ON based on I-DOS score, Tarsitano et al. [62] found the highest prevalence of the disorder in individuals following an omnivorous diet (30.3%), followed by vegan or vegetarian diet followers (23.7%) and individuals adhering to other dietary patterns (20%) [62]. Corresponding observations were made by Dunn et al. [36]. In a group of 275 U.S. college students, authors investigated the prevalence of ON using the ORTO-15 questionnaire. The results showed that 71% of participants scored in the orthorexia range, but only 20% followed restrictive diets. In fact, participants who followed a vegan diet obtained the highest (least pathological) scores. However, it should be noted that this conclusion was drawn based on a very small sample—there were only 6 vegan subjects among 275 study participants [36].

An important aspect of diagnosing ON in vegetarians is the fact that they are more likely to have greater nutritional knowledge and pay more attention to the quality and composition of the products they choose. In a systematic review, in 9 of 12 analyzed studies, lacto-ovo-vegetarians or vegans had a 4.5–16.4-point higher HEI-2010 (Healthy Eating Index 2010) score than non-vegetarians [65]. Additionally, Brytek-Matera et al. [13] showed that vegans had greater knowledge of healthy eating compared to non-vegetarians and vegetarians excluding only meat from their diet. 

Furthermore, the currently used diagnostic methods may not be adapted to vegans and other vegetarians for several reasons. Inherently, these diets require some level of dietary restraint to ensure that unacceptable foods are not consumed. Questionnaires may not be able to distinguish between underlying factors behind the restrictions—whether it is dietary restraint (e.g., plant-based diet) or cognitive restraint (e.g., weight control). In addition, they are likely unable to identify dietary behaviors unique to vegetarians, such as monitoring meal compositions when eating in public or reading food labels (Table 2). Therefore, it seems necessary to design a specific screening tool to assess eating disorder symptoms in individuals who follow plant-based diets. A validated self-report tool dedicated to this group would provide clinicians and researchers with a rapid, inexpensive, and effective way to identify individuals who may require in-depth assessment of dietary habits or intervention [53]. 

Recently, a method for diagnosing eating disorders dedicated to vegans and vegetarians—the Vegetarian Vegan Eating Disorder Screener (V-EDS)—was created. This is an 18-item self-report tool designed to assess eating disorders symptoms in vegetarians and vegans over the past 7 days. It was created based on the experience of vegetarians and vegans, the experience of people with a history of eating disorders, and the knowledge of psychologists and dietitians specializing in the field. The V-EDS was designed to distinguish the basic factors determining the increasing pathology of eating disorders (e.g., restricting food to affect body weight versus restricting food groups in vegetarian or vegan diets). The questionnaire is divided into two parts—the first one, consisting of 6 items, provides information on the respondent’s diet, while the second, a 12-item part, refers to the respondent’s behaviors and attitudes and examines the presence of pathology towards eating disorders. Both are assessed on a 5-point Likert scale, and the higher the obtained score, the more severe the eating disorder. An overall score of 18 is considered as the cut-off point for predicting clinical cases [53,66]. 

The V-EDS has the potential to serve as an accessible tool for the initial screening and ongoing monitoring of eating disorder symptoms progression among vegetarians and vegans. However, this tool has been only preliminarily validated in a small, mainly female sample. Future studies should aim to broaden the psychometric evaluation of the V-EDS by testing it with larger and more diverse populations and considering factors such as gender, age, cultural background, and history of eating disorders [66].

## 8. Conclusions

Based on the available literature, an association between ON and plant-based diets can be observed. However, the lack of consensus on the definition of ON and its diagnosis as well as the small number of studies involving people with a history of eating disorders who also adhere to plant-based diets makes it difficult to demonstrate a clear cause–effect relationship. Studies suggest that vegetarian diets may be chosen as an attempt to cover up orthorexic behaviors, while the dietary restrictions resulting from adopting these diets may potentially predispose individuals to disturbances in eating habits. Thus, it is important for specialists (including dietitians) to pay attention to the motives for choosing such diet types (health aspects vs. weight control) and their patients’ general relationship with food. However, it is important to note that well-planned vegetarian diets can offer numerous health benefits, and the mere fact of adherence to these dietary patterns does not indicate orthorexia. Moreover, potential misinterpretation of the results obtained using existing diagnostic tools may lead to overdiagnosis and stigmatization of plant-based diets users; hence, it is necessary to develop new methods (or adapt current ones) that are more suitable for vegetarians, particularly vegans.

This narrative review has several limitations. It excluded non-English and non-Polish studies, and the overall scarcity of research on this subject hindered the interpretation of the results. Additionally, identifying studies that specifically investigated this issue was difficult; thus, the available literature provides only partial insights, failing to comprehensively explore this relatively new topic or offer definitive conclusions. Furthermore, as a non-systematic review, this study is more susceptible to bias and subjectivity due to the absence of a standardized methodology and a structured search strategy. Further research is needed to explore the relationship between ON and plant-based diets, including understanding of the cause and effect relationship, a clearer definition of ON, and the distinction between HO and ON. However, to our knowledge, this is the first study that offers a holistic perspective on the subject. Our work considers both the potential increased risk of orthorexia associated with plant-based diets and the risk of stigmatization of their adherents. By highlighting these two aspects, we underscore the need for a nuanced approach to assessing orthorexic tendencies in individuals following plant-based diets, ensuring that the diagnostic criteria are both sensitive and specific.

## Figures and Tables

**Table 1:** The most frequently used and best described questionnaires for diagnosing orthorexia nervosa.

Name	Structure	Comment
**EDE-Q** (Eating Disorder Examination Questionnaire)	28 items. Four subscales quantifying: “restraint”, “eating concerns”, “weight concerns”, and “shape concerns” [29].	Reliable and valid measure of eating disorders in diverse samples [29]. Validated in studies of omnivores, weight-motivated vegetarians, and non-weight-motivated vegetarians [30]. Brief three-factor model (7 items) validated in vegans and omnivores [31].
**DEBQ** (Dutch Eating Behavior Questionnaire)	33 items and three subscales. Uses a 5-point Likert scale ranging from never (1) to very often (5). A mean score is calculated for each subscale. Higher scores on each subscale reflect higher levels of restrained, emotional, and external eating, respectively [32].	A tool designed to assess restraint, emotions, and response to external factors of eating. Good psychometric properties of eating disorder scales in vegans [33].
**ORTO-15**	15 items. Answers indicating the risk of orthorexia behavior receive 1 point, while those corresponding to a correct attitude to food receive 4 points. The cut-off point is 40 points. Obtaining a lower number of points indicates a tendency toward ON. This test and its adaptations are most often used in scientific research [34].	Does not detect true pathological eating behaviors but rather normal dietary adherence in the vegetarian/vegan populations [35]. Overestimates the prevalence of ON. Poor psychometric properties. Probably cannot distinguish between ON and HO [36].
**TFEQ** (Three-Factor Eating Questionnaire)	18 items. Assesses three cognitive and behavioral domains of eating: restrained eating, binge eating, and emotional eating. There is no universally used cut-off point [37].	Suitable rather for describing eating habits than for diagnosing eating disorders [37].
**EAT** (Eating Attitudes Test)	40 items. 6-point Likert-type self-rating scale. Individuals with a total score of 30 or higher are considered to have disordered eating [38].	Poor psychometric properties in vegans [38].
**EHQ** (Eating Habits Questionnaire)	21 items. Based on the 4-point Likert-type scale (from “false, not at all true” to “very true”). The areas included in the questionnaire are knowledge about healthy eating, problems related to healthy eating, and positive attitudes towards healthy eating [39].	Detects both ON and HO, with inability to distinguish between these separate constructs [40].
**BOT** (The Bratman Orthorexia Test)	10 items. A score of 0–1 point means that the examined person does not suffer from ON, 2–3 points indicate a risk of this disorder, and 4 or more is diagnosed as ON [25].	Not a psychometric test [25].
**DOS** (Düsseldorf Orthorexia Scale)	10 items. Based on a 4-point Likert-type scale (from “this does not apply to me” to “this applies to me”) [41]. Cut-off point is 30, while scores ranging from 25 to 29 indicate a risk of ON [41].	Inability to distinguish between patients suffering from anorexia and orthorexia [42]. Detects both ON and HO, with inability to distinguish between these separate constructs [40].
**TOS** (Teruel Orthorexia Scale)	Based on the 4-point Likert-type scale (from “does not concern you at all” to “completely affects you”). Includes two subscales—TOS He (9 items) and TOS Ne (8 items)—for diagnosing HO and ON, respectively. A higher score on both scales indicates a greater intensity of a given form of orthorexia [43].	Extends the conceptualization of orthorexia beyond the problematic aspects of healthy eating to include non-problematic ones, i.e., HO. Operates independently of any underlying psychopathology [42].

**Table 2:** Selected problematic and potentially misleading elements for vegetarians, with emphasis on vegans, in questionnaires for diagnosing orthorexia nervosa. Based on [47].

Name of Questionnaire	Problematic and Potentially Misleading Elements for Vegetarians with Comments
**EDE-Q**	Questions 3 and 4 on the restraint scale: **Have you tried to eliminate foods you like from your diet to affect your figure or weight?** **Have you tried to follow certain rules about food to affect your figure or weight?** Vegetarians exclude some or all animal products from their diet.
**ORTO-15**	Questions 2 and 8: **Do you feel confused when you go to the grocery store?** Vegans can often feel confused when reading the list of ingredients and assessing whether a product contains animal-derived ones. **Do you allow yourself any food-related transgressions?** Vegans usually do not allow themselves to consume animal products. However, it is important to remember that these limits are very individual.
**TFEQ**	Item 1 on the subscale of cognitive inhibition: **When I smell a sizzling steak or see a juicy piece of meat, I find it very difficult to stop eating, even if I have just finished a meal.** Vegetarians do not eat meat; thus this question seems inadequate and the answer unreliable.
**EAT**	Items 2, 19, 30, 32, and 33: **You prepare food for others, but you will not eat it yourself.** Vegans can often prepare dishes from animal products for others. **You like eating meat.** Vegetarians do not eat meat; thus this question seems to be inadequate and the answer unreliable. **You eat diet foods.** Products for vegetarians are often considered “diet” foods. **You show self-control when dealing with food.** Vegans must show self-control when dealing with foods containing animal products. **I feel that others put pressure on me to eat.** Vegans may feel pressured by family and friends to eat animal products because of a lack of understanding of their choices.
**YFAS** (Yale Food Addiction Scale)	Items 11 and 23 on the subscale of important social, work, or recreational activities that have been cut back or are being cut back: **There were times when I avoided work or social situations because I was unable to eat certain foods there.** Vegans may avoid work or social situations where vegan food options are limited. **I have tried to limit my consumption of certain foods.** Vegetarians limit some or all animal products. Item 24 on the subscale of persistent craving or repeated failed attempts to quit: **I have managed to limit or not eat these types of foods.** Vegetarians limit some or all animal products.
